# Benchmarking commercially available soft and rigid passive back exoskeletons for an industrial workplace

**DOI:** 10.1017/wtc.2024.2

**Published:** 2024-02-15

**Authors:** Mohamed I. Mohamed Refai, Alejandro Moya-Esteban, Lynn van Zijl, Herman van der Kooij, Massimo Sartori

**Affiliations:** Department of Biomechanical Engineering, University of Twente, Enschede, The Netherlands

**Keywords:** biomechanics, exoskeletons, exosuits

## Abstract

Low-back pain is a common occupational hazard for industrial workers. Several studies show the advantages of using rigid and soft back-support passive exoskeletons and exosuits (exos) to reduce the low-back loading and risk of injury. However, benefits of using these exos have been shown to be task-specific. Therefore, in this study, we developed a benchmarking approach to assess exos for an industrial workplace at Hankamp Gears B.V. We assessed two rigid (Laevo Flex, Paexo back) and two soft (Auxivo Liftsuit 1.0, and Darwing Hakobelude) exos for tasks resembling the workplace. We measured the assistive moment provided by each exo and their respective influence on muscle activity as well as the user’s perception of comfort and exertion. Ten participants performed four lifting tasks (*Static* hold, *Asymmetric*, *Squat*, and *Stoop*), while their electromyography and subjective measures were collected. The two rigid exos provided the largest assistance during the *Dynamic* tasks. Reductions in erector spinae activity were seen to be task-specific, with larger reductions for the two rigid exos. Overall, Laevo Flex offered a good balance between assistive moments, reductions in muscle activity, as well as user comfort and reductions in perceived exertion. Thus, we recommend benchmarking exos for intended use in the industrial workplace. This will hopefully result in a better adoption of the back-support exoskeletons in the workplace and help reduce low-back pain.

## 1. Introduction

Low-back pain (LBP) is a frequently occurring occupational hazard for industrial workers (Pesenti et al., 2021). The lifetime prevalence of LBP for people in industrialized countries is 60–70% according to the World Health Organization (Lodato and Kaplan, 2013). This translates to high healthcare costs. The total cost for LBP-induced leave in the Netherlands was estimated to be 244.7 million euros in 2017 (van der Wurf et al., 2021). Chronic LBP influences psychosocial factors resulting in low quality of life (Járomi et al., 2021; Tom et al., 2022). Although the etiology of LBP is not completely understood, possible causes are injuries to muscles, tendons, and intervertebral disks of the spine (Wai et al., 2010).

Occupational risk factors for LBP include heavy lifting, repetitive heavy work, and working in non-ergonomic postures (Da Costa and Vieira, 2010). Ergonomic guidelines set by the Revised NIOSH Lifting equation recommend a maximum limit of 3.4 kN of disk compression force on the lumbosacral joint during lifting activities at the workplace, especially when standing upright with the load (Waters et al., 1993). However, in practice, the compressive forces, which influence the risk of developing LBP, are much higher (Ali et al., 2021; Moya-Esteban et al., 2022).

Current measures to prevent LBP include improving workplace ergonomics, creating awareness about lifting techniques, and the use of cranes or jibs for assistance. Nevertheless, prolonged lifting could still lead to LBP despite using ergonomic postures. Furthermore, users may find cranes to be expensive, slow, and bulky. Wearable assistive devices are potential alternatives that help reduce risks of LBP without hindering movement or offering discomfort (Lamers et al., 2018; Ali et al., 2021; De Bock et al., 2022). This includes passive back-support rigid exoskeletons or soft exosuits (collectively, *exos*) that are targeted to aid a person in executing a specific task (Lowe et al., 2019; Pesenti et al., 2021; De Bock et al., 2022). Exos help reduce physical effort and alleviate the musculoskeletal system by reducing muscle activity, and thereby spinal loads (especially at L5/S1 joint). Therefore, they have enormous potential to reduce LBP within occupational settings (Lamers et al., 2018; Ali et al., 2021; Pesenti et al., 2021).

Back-support rigid exoskeletons use rigid structures to provide forces perpendicular to the human–machine interface to generate a back-extension moment wear (de Looze et al., 2016), while soft exosuits use elastic or pneumatic components to provide forces parallel to the trunk (Toxiri et al., 2019). Consequently, rigid exos are generally bulkier, heavier, and more difficult to wear compared to soft exosuits.

Several studies in literature have assessed exos extensively on their objective and subjective impact on the user (Toxiri et al., 2019; Del Ferraro et al., 2020; Kermavnar et al., 2021; Pesenti et al., 2021; De Bock et al., 2022; Kang and Mirka, 2023a). Objective assessments show benefits in improved range of motion (Schmalz et al., 2022; Reimeir et al., 2023) and reductions in muscular effort (Thamsuwan et al., 2020; Luger et al., 2021; Schwartz et al., 2021; Schmalz et al., 2022; Kang and Mirka, 2023a, 2023b), total metabolic cost or energy expenditure (Del Ferraro et al., 2020; Erezuma et al., 2022; Schmalz et al., 2022), and low-back compressive forces (Koopman et al., 2019a, 2019b; Schmalz et al., 2022). Although studies found overall positive outcomes on subjective assessment of safety, comfort, ease of use, usability, and perceived rate of exertion (Baltrusch et al., 2018; Omoniyi et al., 2020; Flor et al. 2021), these assessments were found to be user-dependent (Omoniyi et al., 2020). For instance, localized discomfort in wearing the exos was a concern (Bosch et al., 2016; Alemi et al., 2020; Madinei et al., 2020). Finally, a few studies incorporated both objective and subjective measures of the exo on the user showing that exos reduced peak trunk extensor muscle activity, energy expenditure, and perceived lower exertion (Bosch et al., 2016; Alemi et al., 2020; Madinei et al., 2020; Erezuma et al., 2022).

Although overall positive, studies have shown that the effects of exos depend on the device design, user, and task (Alemi et al., 2020; Thamsuwan et al., 2020; De Bock et al., 2021). For instance, one study reported that users with existing LBP demonstrated more benefits from using an exo than those without existing LBP (Ulrey and Fathallah, 2013). Additionally, some studies highlight muscle activity reduction (Graham et al., 2009; Motmans et al., 2019), while others report higher user discomfort (Amandels et al., 2019). Furthermore, some studies have found that benefits of exos may not translate directly to the field (De Bock et al., 2021). To address this, there are adoption frameworks available to help assess which exo was suitable for an intended task at a given workplace (Ashta et al., 2023; Golabchi et al., 2023). Thus, there is a need for assessing exos using a workplace-specific benchmark.

Thus, in this study, we aim to set a multimodal benchmark to assess the impact of exos on workers at a gear manufacturing company Hankamp Gears B.V. We scouted the handling of the Computer Numeric Control (CNC) milling machine workplace and simulated the working conditions at the laboratory. Four passive back-support exos; two rigid (Laevo Flex [Laevo B.V., Rijswijk, The Netherlands] and Paexo back [Ottobock SE & Co. KGaA, Duderstadt, Germany]), and two soft (Darwing Hakobelude [Daiya Industry Co., Ltd., Okayama, Japan] and Auxivo Liftsuit 1.0 [Auxivo AG, Schwerzenbach, Switzerland]) were tested. The benchmark incorporated both objective and subjective measures. This included the apparent assistive moment provided by each exo, changes to the participant’s muscle activity as a function of this support, and their perceived exertion and subjective comfort when using the device.

## 2. Methods

The participant information and measurement setup are described in Section 2.1. Section 2.2 describes the four exos tested and the estimation of the assistance provided by them during the tasks performed. Section 2.3 describes the experimental procedure, and data processing is described in Section 2.4. Finally, the analysis performed is summarized in Section 2.5.

### 2.1. Participants and measurement setup

Ten healthy (5 men, 5 women, age: 23 ± 1.8 years, weight: 70 ± 6.7 kg, height: 1.82 ± 0.07 m) with no prior history of exo use participated in the study. This convenience sample was not based on a priori sample size calculations. All participants gave written informed consent. All participants reported no current back injuries or other injuries preventing them from naturally performing the tasks. Ethical approval was obtained from the University of Twente (reference 2022.134).

**Figure 1.**
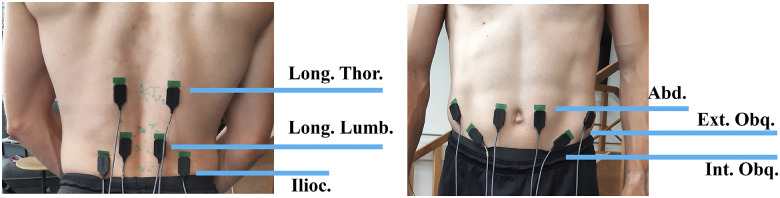
Bilateral EMG electrodes were placed on the Longissimus Thoracis (Long. Thor.), Longissimus Lumborum (Long. Lumb.), Iliocostalis (Ilioc.), the Rectus Abdominus (Abd.), Internal Obliques (Int. Obq.), and the External Obliques (Ext. Obq.).

Muscle activity was collected using Delsys Bagnoli (Delsys, Boston, MA). Surface electromyography (EMG) data were collected from six bilateral muscles (Figure 1). This included the abdominal rectus abdominis (Abd.) at the umbilicus level, internal obliques (Int. Obq.), external obliques (Ext. Obq.), longissimus lumborum pars lumborum (Long. Lumb.) placed 6 cm lateral to L2, longissimus lumborum pars thoracis (Long. Thor.) placed 4 cm lateral to T10, and Iliocostalis Lumborum (Ilioc.) at 3 cm lateral to L1 (Figure 1; Moya-Esteban et al., 2020). The bipolar EMG electrodes were placed both on left and right sides of the trunk and were sampled at 2048 Hz. An optical motion capture system (Qualisys Medical AB, Gothenburg, Sweden) was used to capture the trajectories of reflective markers placed on the body and the exos. Participants had markers placed at the C7 and T10 vertebrae, clavicle, both acromions and anteriorly and posteriorly on the sacroiliac joint. Markers were also placed on the exos (Supplementary Appendix A), which included the exo hip joint and thigh pad for the rigid exos, and either side of the elastic bands for the soft exos. The trajectories were sampled at 128 Hz.

### 2.2. Exoskeletons used and assistance provided

The four exos used in this study are detailed in Table 1. Exo fitting was based on specifications provided by the manufacturer and on subjective user comfort while aiming for maximum assistance. Paexo’s back assistance was set to the static (maximal assistance) mode. Laevo suggests a sizing chart to estimate the stiffness of the gas spring used in the exo; however, to ensure similar assistance across all participants, the medium stiff gas spring (42 kN/m) of the Laevo Flex was used.

**Table 1.** Exoskeletons and exosuits used in the study

	Laevo Flex	Paexo back	Darwing Hakobelude	Auxivo Liftsuit 1.0
Short name	Laevo	Paexo	Darwing	Auxivo
Company	Laevo B.V.	OttoBock SE & Co. KGaA	Daiya Industry Co.	Auxivo AG
Weight (kg)	4.3	4.4	0.8	0.9
Type	Rigid	Rigid	Soft	Soft
	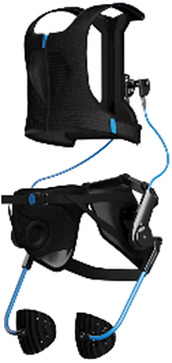	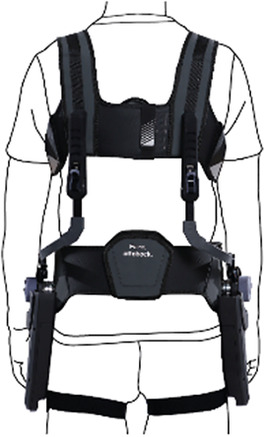	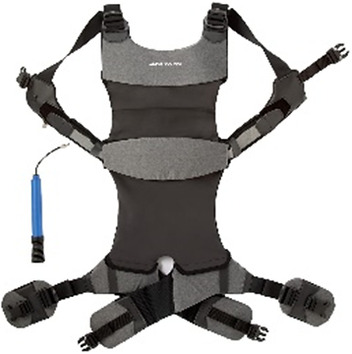	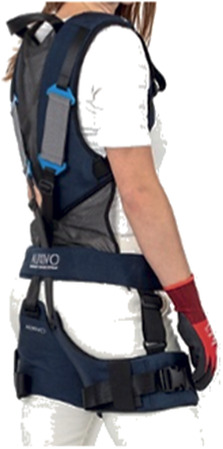

#### 2.2.1. Apparent assistance provided by the exos used

The apparent assistance provided by each exo around the sacrum was identified by estimating the forces exerted by the exos on the trunk during the *Dynamic* task. This was estimated by using two different approaches for each exo type (rigid vs. soft) (Supplementary Appendix A).

**Figure 2.**
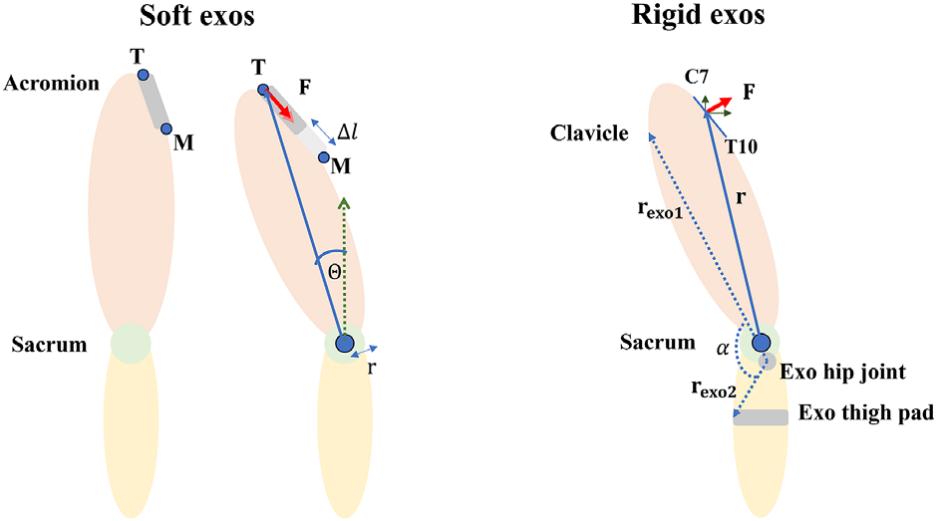
Estimation of the apparent assistive moments ( 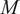 ) exerted by the soft and rigid exos. *Soft exos*: Forces due to elongation of the elastic bands ( 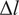 ) were estimated using a predetermined force–elongation relation. Sagittal 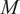 was estimated by scalar multiplication of forces with a constant moment arm 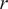 (10 cm)*. Rigid exos*: Trunk forces ( 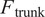 ) were estimated from the moment exerted by the exo ( 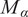 ). 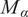 was estimated using the exo angle ( 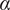 ) (Van Harmelen et al., 2022). The forces exerted were resolved to their 3D components ( 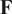 ). The sagittal apparent assistive 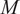 was derived using the cross-product of the moment arm ( 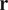 ) and 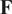 . Trunk inclination ( 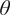 ) for both rigid and soft exos was defined as the inclination of a line from the sacrum to the acromions with respect to the vertical axis.

The apparent assistance provided by the two soft exos was estimated by first defining the force–elongation relation (Supplementary Appendix A). The influence of back bending and, therefore, curvature in the elastic band were assumed to be small. During *Dynamic* tasks, the elongation ( 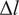 ) of the elastic band was measured as the change in length between markers **T** and **M** (Supplementary Figure A.1) that were placed at either end of the elastic band as
(1)

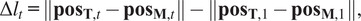

where 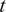 denotes the time instance during the *Dynamic* tasks. Two fits were identified, one for loading, and the other for unloading of the elastic bands. We assumed that during flexion, the elastic bands are loaded, and during extension, they are unloaded. This required segmenting the *Dynamic* tasks into flexion and extension movements. For this, the velocity of the trunk inclination ( 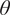 ) (Figure 2) was estimated. First, 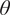 was defined as the inclination of the vector ( 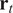 ) between the acromion and sacral markers with respect to the vertical axis. Then, the direction of the velocity of 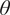 was used to segment the *Dynamic* tasks. During flexion and extension, the loading and unloading force–elongation relations were, respectively, used to estimate the forces ( 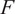 ) exerted by the elastic bands. 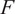 was multiplied with a constant moment arm of 10 cm to estimate the sagittal moments 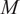 (Lamers et al., 2018; Rodzak et al., 2023).

The apparent assistance provided by the two rigid exos was estimated by using the torque–angle relationship measured using the setup at Laevo B.V. (Van Harmelen et al., 2022). Polynomial fits were applied to these torque–angle curves (Supplementary Appendix A). During *Dynamic* tasks, we estimated the exo angle ( 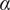 ) as the angle between two vectors 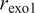 and 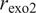 (Figure 2; Van Harmelen et al., 2022). 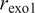 was defined as the vector between the marker placed on the clavicle and the exo hip joint (axis of rotation for the exo). 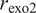 was defined as the vector between the markers placed on the exo hip joint and the exo thigh padding. The direction of 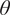 velocity was used to segment *Dynamic* tasks into flexion and extension movements. Thus, the corresponding loading torque–angle curve was used during flexion and extension. This provided an instantaneous exo moment ( 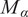 ) which was used to find the forces acting on the trunk ( 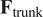 ) (Supplementary Appendix A). These 3D forces were used to estimate the apparent assistive moments around the sacrum using 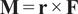 at each time instant 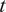 . The sagittal moments are then plotted against 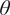 .

### 2.3. Experimental procedure

**Figure 3.**
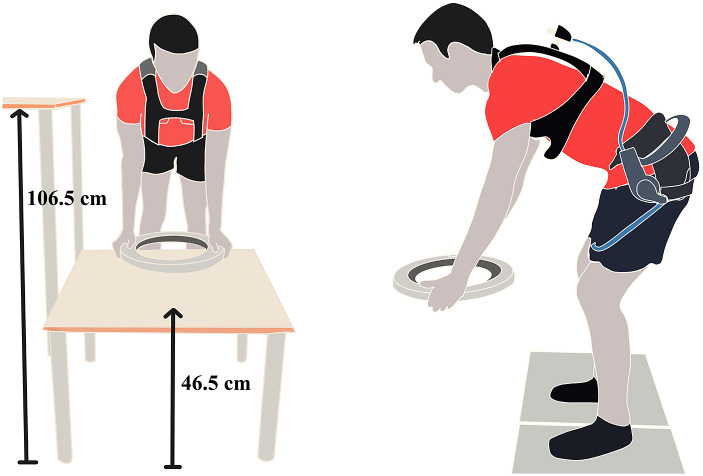
Experimental setup. Left: Front view of the setup, with user wearing the Darwing. Right: Side view of the setup, with user wearing Laevo.

A researcher shadowed a worker’s daily routine at the CNC workplace (Supplementary Figure B.1) at Hankamp Gears B.V. The workers loaded gears from a box into the machine for milling. After milling, they placed it temporarily on another table and then stacked it into a box for delivery. The heights of the milling machine and the table were measured. These measurements were used to replicate the experimental setup at our laboratory with two tables 46.5 and 106.5 cm tall (Figure 3). The gear (weighing 12.6 kg) milled at the CNC machine was borrowed from Hankamp Gears B.V. for the experimental protocol. Based on the worker’s daily routine, four tasks were identified. They were standardized into the following tasks for the experimental protocol:

1. *Static*: Participants were instructed to stoop at two predefined trunk inclination angles of 40° and 60°. Trunk inclination was measured with an inertial measurement unit on the participants’ back at the T10 level, and visual feedback was provided on a laptop. Before measurements, participants were asked to bend as far as possible to determine if they could achieve 90°, thereby preventing the flexion relaxation phenomenon (Kippers and Parker, 1984).
2. *Asymmetric*: Participants were asked to lift the gear from the low table in front to the high table placed on the right (Figure 3). The participants then assumed a neutral erect standing posture before lifting the gear from the high table and returning it to the low table.
3. *Squat*: Participants performed squats by using minimal back bending and as much knee flexion as possible to lift the gear from the low table. After standing up straight, they placed it back on the table. This task was not performed with the Paexo back as it did not allow this degree of freedom.
4. *Stoop*: Participants stooped by flexing the trunk as much as possible and with minimal knee flexion (avoiding locked knees) to lift the gear from the low table. After standing up straight, they placed the gear back on the table.

A snapshot of a participant performing these tasks is provided in Supplementary Appendix B. The *Asymmetric*, *Squat*, and *Stoop* tasks are referred to together as *Dynamic* tasks. The *Asymmetric* task required movement in all three anatomical planes, whereas the *Squat* and *Stoop* tasks required movement only in the sagittal plane. Five sets of two repetitions were performed for the *Asymmetric*, *Squat*, and *Stoop* tasks. The participants performed the lifting task phases following a metronome at 30 bpm. In the case of the *Static* task, the participants were asked to perform it three times, each 5 s long.

After each task, the participants were asked to rate their perceived rating of exertion (PRE) based on the 15-point Borg scale (scale B) (Borg, 1973). The lowest value was 6 corresponding to “no sense of effort,” and the highest possible value was 20 which represented “maximum” effort by the participants. After completing all trials with one exo, participants filled in a visual analogue discomfort scale (Wewers and Lowe, 1990). This questionnaire assessed the discomfort caused by the exo at different parts of the body. The participants selected a point along a 10 cm line that ranged from 0 (no discomfort) to 10 (maximum discomfort). The distance of this point from the start was measured and taken as the discomfort experienced. The participants were all Dutch and used the Dutch version of both RPE and VAS. The Dutch and English versions of these scales are provided in Supplementary Appendix C.

All tasks were performed while wearing the exo as well as without the exo (no-exo condition). The order of exos and tasks was randomized.

### 2.4. Data processing and analysis

The raw EMGs were first bandpass-filtered between 30 and 300 Hz. Then, the signal was rectified and low-pass-filtered (cutoff frequency of 6 Hz) to find the linear envelope. The EMG amplitude was normalized to the maximum voluntary contraction (MVC) of the specific muscles. MVC targeting each muscle group was obtained during two trials in which the participants maximally activated the respective muscle against resistance provided by the researcher. All filters used were fourth-order zero-lag Butterworth filters.

The root mean square (RMS) of the EMG amplitude was computed for the *Static* trials. The apparent assistive moments provided by the four exos during the *Dynamic* tasks were estimated and averaged across participants. We measured the moments only for the first lifting cycle, which began with the first flexion to pick up the gear and then extended with the gear to a neutral standing position. The moments were plotted against 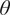 . Loss of energy during lifting was measured by subtracting the area under the moment–angle curve between the flexion–extension phases of the *Dynamic* task. This provided the hysteresis for each of the four exos. Then, the integral of EMG amplitude (iEMG) was calculated for the *Asymmetric*, *Squat*, and *Stoop* trials to estimate differences in overall muscle effort across trials. The iEMG was estimated only when the participant was moving, which was identified using the hip flexion velocity.

### 2.5. Data analysis

Statistical analysis was done using SPSS version 28.0.1.0 (IBM Corp, USA) and MATLAB version 2019b (Mathworks, USA). Test for normality of distributions was performed using the Shapiro–Wilk test. Distributions of iEMG, RMS, and subjective questionnaire outcomes were found to be not normally distributed. Hence, differences between exo conditions were analyzed using the independent-samples Kruskal–Wallis test, followed by a post hoc test. The distribution of global discomfort scales was normally distributed, and therefore, ANOVA with the post hoc Tukey test was used to determine differences between exo conditions. Independent-samples Mann–Whitney test was used to test differences for the global discomfort scale across genders.

## 3. Results

Due to sensor issues, EMG data for one participant wearing the Darwing were removed from further analysis. Changes in muscle activity for the Abdominal, Iliocostalis, and Longissimus Lumborum are only shown here. The changes in activity for the other muscles (Internal and External Obliques, and Longissimus Thoracis) are shown in Supplementary Appendix D.

**Figure 4.**
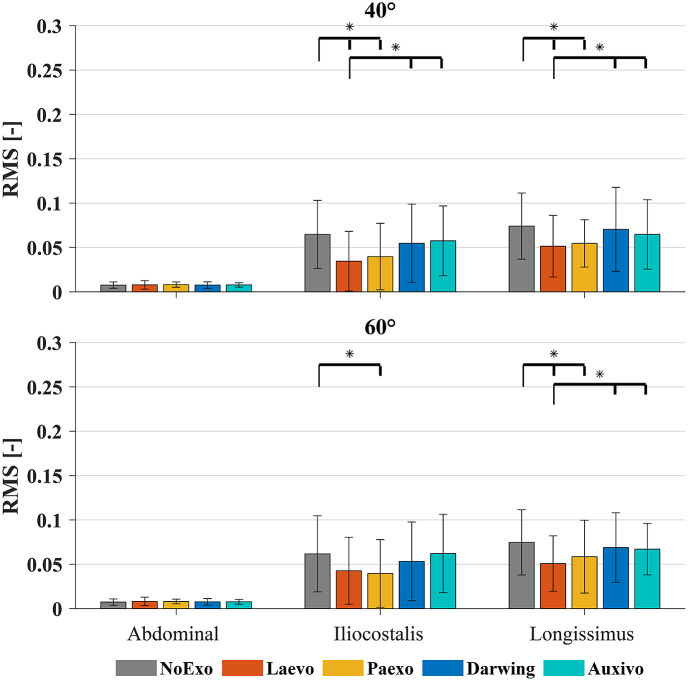
Median RMS of the normalized EMG during the *Static* task across all participants. The Longissimus Lumborum muscle activity is shown here. Error bars represent the interquartile range. Significant differences are represented by the horizontal bars with *. The differences are read with respect to the leftmost condition (marked by the long thin line pointing down), and those that follow (shorter line pointing down).

The median RMS of the normalized EMG amplitude across participants for the *Static* task is seen in Figure 4. Post hoc Bonferroni correction for multiple comparisons was applied. For both inclination angles, significant reductions (*p* < .05) in muscle activity were found between no exo and either rigid exos for the Longissimus muscle. Significant differences were also seen between Laevo and both soft exos for the Longissimus muscle.

**Figure 5.**
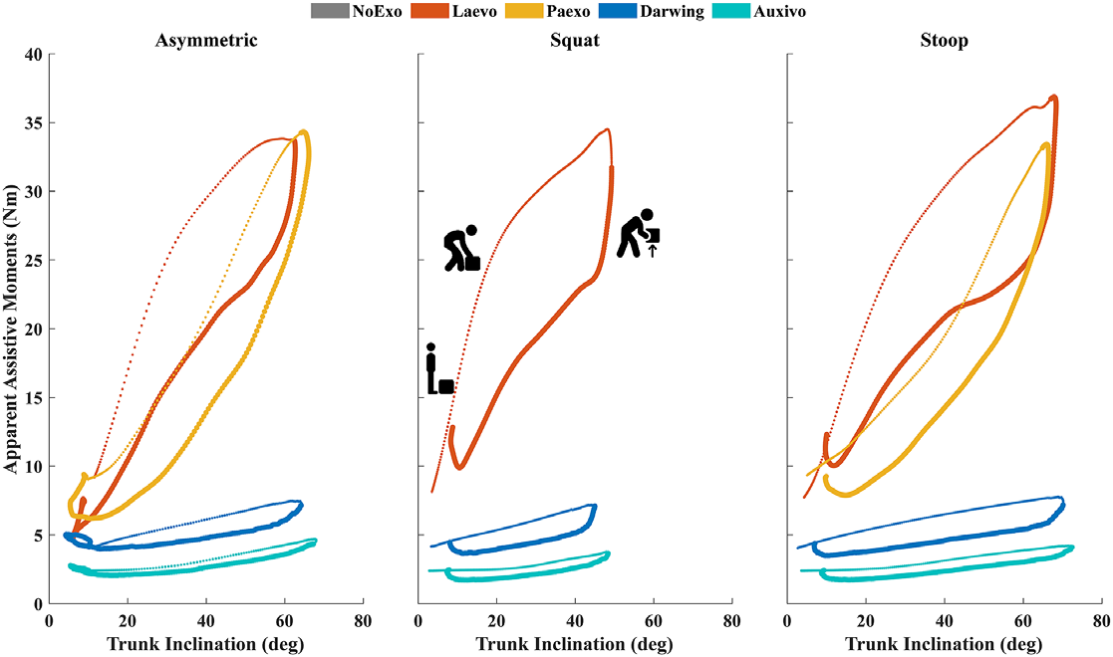
Average apparent assistive moment–angle curves provided by the four exos across all participants and tasks during the first lifting cycle. The cycle includes flexing down to lift the gear (denoted by thin dots) and extending to stand upright (denoted by thicker dots). Snapshots of the lifting phases are shown for the Laevo curve during *Squat.*

Figure 5 shows the differences in support provided by the four exos across the *Dynamic* tasks, and Table 2 shows the hysteresis of the different exos for each lifting trial.

**Table 2.** Hysterisis (Nmrad) from Figure 5

	Asymmetric Nmrad (%)	Squat Nmrad (%)	Stoop Nmrad (%)
Laevo	−6.8 (−29.2)	−7.9 (−37.9%)	−9.8 (−33.9%)
Paexo	−6.3 (−32.4)	–	−6.1 (−30.9%)
Darwing	−1.0 (−18.6)	−1.3 (−30.9)	−2.2 (−30.3)
Auxivo	−0.5 (−15.8)	−0.7 (−30.3)	−1.1 (−28.1)

*Note.* The percentage difference with respect to lowering phase is shown within parentheses.

**Figure 6.**
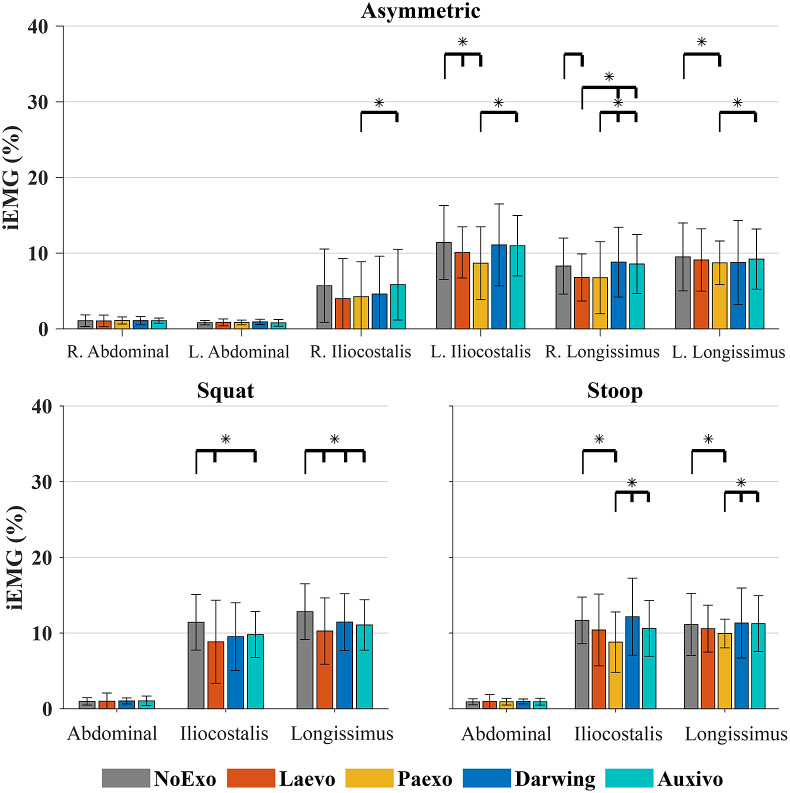
Median integral EMG of the *Dynamic* tasks across all participants. The Longissimus Lumborum muscle activity is shown here. Error bars represent the interquartile range. Significant differences are represented by the horizontal bars with *. The differences are read with respect to the leftmost condition (marked by the long thin line pointing down), and those that follow (shorter line pointing down).

Figure 6 shows the median iEMG across the participants for the *Asymmetric*, *Squat*, and *Stoop* tasks. The left and right muscles are denoted separately for the *Asymmetric* task. For the *Squat* and *Stoop* conditions, the muscle activity on either side is averaged. Post hoc Bonferroni correction for multiple comparisons was applied.

During the *Asymmetric* task, Laevo showed significant reductions in muscle activity with respect to the no-exo condition for the left Iliocostalis and right Longissimus, whereas Paexo showed reductions in left Iliocostalis and on both sides of the Longissimus. Neither of the soft exos showed significant reductions with respect to the no-exo condition. Differences among the four exos varied and were muscle-specific. For instance, Laevo was different with respect to either soft exo only when considering the right Longissimus muscle activity. Paexo showed differences with the Auxivo for three muscles including both sides of the Longissimus and left Iliocostalis. Paexo also had significant reductions compared to the Darwing when considering the right Longissimus muscle.

The three exos tested in the *Squat* task showed significant reductions with respect to no-exo condition for the Longissimus muscle activity. Additionally, Laevo and Auxivo also reduced the Iliocostalis muscle activity when comparing no-exo condition. Finally, for the *Stoop* task, among the two rigid exos, only Paexo showed significant reductions for the erector spinae muscles with respect to the no exo as well as either soft exo. The changes in muscle activity are compared with the assistive moments provided by each exo in Supplementary Appendix E.

The rigid exos or soft exos did not show differences in muscle activity among themselves (i.e., Paexo vs. Laevo and Auxivo vs. Darwing) for any of the *Dynamic* tasks. Finally, the reductions in abdominal muscle activity were not significant for any of these tasks.

Table 3 shows the results of the discomfort scale. Laevo and the Darwing were considered to be the most comfortable among the four tested. The most discomfort for Auxivo and Paexo was around the legs. Differences between global discomfort scores between men and women were not significant (*U* = 172, *p* = .46). Table 4 shows that for three out of four tasks, Laevo showed the lowest perceived exertion. However, only the *Asymmetric* task showed a significant (*p* < .05) difference from no-exo condition. Although the *Static* task had the lowest scores for perceived exertion across tasks, all exos except Paexo had a higher exertion than the no-exo condition, though the differences were not significant.

**Table 3.** Discomfort scale averaged across participants

	Global	Chest	Upper back	Lower back	Abdomen	Front legs	Back legs
Laevo	1.7 ± 1.7	0.8 ± 1.3	0.9 ± 1.0	0.5 ± 0.8	0.4 ± 1.3	1.6 ± 2.1	0.8 ± 1.4
Paexo	3.3 ± 1.7	2.3 ± 1.9^a^	1.4 ± 2.0	0.9 ± 1.4	1.4 ± 1.7^a^	2.4 ± 2.3	1.7 ± 1.7
Darwing	1.6 ± 0.8	2.0 ± 1.9	1.7 ± 2.1	0.4 ± 0.6	0.0 ± 0.1^b^	1.0 ± 1.1	0.9 ± 0.8
Auxivo	3.5 ± 1.5^a^ ^,^^c^	1.6 ± 1.2	1.0 ± 1.2	0.4 ± 1.0	0.8 ± 1.0	3.0 ± 2.8	3.6 ± 2.4

*Note.* The scale ranges from 0 to 10, with 0 corresponding to no discomfort and 10 corresponding to maximal discomfort.

a Significantly different from Laevo.

b Significantly different from Paexo.

c Significantly different from Darwing.

**Table 4.** Perceived rating of exertion averaged across participants

PRE	Static	Asymmetric	Squat	Stoop
No Exo	7.5 ± 1.3	13.9 ± 1.8	12.5 ± 1.8	12.7 ± 2.5
Laevo	7.7 ± 1.7	12.2 ± 1.4^a^	11.1 ± 1.8	11.3 ± 1.7
Paexo	7.4 ± 1.6	12.8 ± 1.4	–	12.3 ± 2.2
Darwing	7.9 ± 1.4	13.6 ± 1.8 2	12.5 ± 2.0	13.9 ± 2.5^b^
Auxivo	7.9 ± 1.4	13.3 ± 1.3	12.2 ± 1.9	12.3 ± 2.1

*Note.* The scale ranges from 6 to 20, with 6 corresponding to no perceived effort and 20 corresponding to maximum effort.

a Significantly different from No Exo.

b Significantly different from Laevo.

## 4. Discussions

In this article, we designed a benchmark to assess the impact of four passive back-support exos for a given industrial workplace. The benchmark assessed Laevo, Paexo, Darwing and Auxivo, for their objective (apparent assistance provided and muscle activity reduction) and subjective (discomfort and perceived exertion) impact on participants performing four tasks simulating the CNC milling machine workplace at Hankamp Gears B.V.

Reductions in muscle activity were found to be task and muscle-specific. For instance, for both *Static* tasks, only Paexo significantly reduced the erector spinae muscle activity compared to the no-exo condition. Laevo was effective for reducing Longissimus activity in both tasks compared to the no-exo condition as well as either soft exo. The exo showed similar results for the Iliocostalis only for the *Static* 40° task. Laevo demonstrated the largest reductions in median EMG, which was on average 38.8 and 31.3% for the Iliocostalis and Longissimus, respectively. Similar reductions were found by the current (Schnieders et al., 2023), and earlier versions of Laevo (Bosch et al., 2016; Baltrusch et al., 2018; Alemi et al., 2019, 2020; Koopman et al., 2019a; Thamsuwan et al., 2020; Luger et al., 2021). Laevo remains to be the most commonly studied back exo in literature (Ashta et al., 2023).

The remaining exos (Paexo, Darwing, and Auxivo) showed a reduction in iliocostalis muscle activity (37.2, 14.6, and 5.2%, respectively) and longissimus muscle activity (23.9, 6.4, and 11.4%, respectively), whereas reductions in Longissimus muscle activity were found to be 23.9, 6.4, and 11.4% for the Paexo, Darwing and Auxivo, respectively. The reductions in the two rigid exos were similar to other exos tested, where an average of 36% reduction in erector spinae muscle activity was found when using Spexor, Reconfigurable Trunk, or HeroWear Apex when performing a *Static* hold task (Lamers et al., 2018; Baltrusch et al., 2020; Gorsic et al., 2020; Wei et al., 2020; Kermavnar et al., 2021; Kang and Mirka, 2023a).

However, these findings contradict the findings of Schwartz et al who showed that the reductions in muscle activity were related to trunk inclination (Schwartz et al., 2021). They found no significant reductions of the erector spinae muscle when wearing a rigid exo and performing a *Static* task with a trunk inclination of less than 75°. The main difference with their study is that they tested an older version of the Laevo (Laevo V1), and the participants carried a lighter load (8 kg). This further strengthens the argument for assessing exos by benchmarking them to the intended industrial task.

Figure 5 shows the average apparent assistive moments provided by the exos around the sacrum during the *Dynamic* tasks. The two rigid exos provided the largest assistance during *Dynamic* tasks (Figure 5). This corresponded to a larger resistance during the lowering phase. Table 2 shows that all exos lose energy due to hysteresis when lifting. On average, the Laevo had a loss of 33.7% which was the same as reported by the manufacturer (at 70% spring strength) (Van Harmelen et al., 2022). Across all trials, the Laevo had a maximum moment arm of 37.4 cm in the sagittal plane. Peak assistance was provided during the *Stoop* tasks (36.9 Nm) when participants were about to lift the gear (Supplementary Appendix E). This was lower than the maximum possible assistance provided by the exo as specified by the manufacturer (41 Nm) for the spring support used. The Paexo had a maximum moment arm of 38.2 cm across trials. The exo offered a maximum support of 34.3 Nm during the *Asymmetric* task which was lower than the maximum possible (49 Nm), whereas the average hysteresis of 31.7% was similar to reported values (Van Harmelen et al., 2022). These differences are most likely due to the differences in operating ranges for the two exos during the tasks. The torque–angle curves reported in the literature show that Laevo provides the highest assistance between 40^o^ to 60^o^ of exo flexion, whereas for the Paexo, peak assistance occurs after 80^o^ (Van Harmelen et al., 2022). Note that Figure 5 depicts the trunk inclination ( 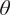 ) on the *x*-axis and not the exo angle ( 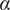 ) (Van Harmelen et al., 2022).

The findings of Auxivo were different from the values reported in literature (Van Harmelen et al., 2022). For instance, the maximum assistance provided was much lower (4.7 Nm) than reported (24 Nm for low tension) (Van Harmelen et al., 2022). Moreover, the hysteresis measured (26.6%) was lower than reported (45%) (Van Harmelen et al., 2022). We saw that the Darwing provided slightly larger assistances, with the highest during the *Stoop* task (7.8 Nm). The average hysteresis for this exo was 24.7%. Differences in the two soft exos could be due to differences in the elongation of the elastic bands during the tasks, the stiffness of these bands, and differences in exo design. Although the Auxivo has a higher stiffness (Supplementary Appendix A), the maximum elongation of the elastic bands of Auxivo and Darwing was 8.3 and 20.8 mm, respectively, during the *Dynamic* tasks. Furthermore, the elastic bands of the Darwing are positioned such that they span from the shoulder blade to the lumbar region. The Auxivo, on the other hand, has elastic bands that are positioned over the shoulder blades, resulting in differences in elongation between the two soft exos. Moreover, the differences in elongation could also be due to slipping of the exos against the participants. Subsequently, Auxivo has been shown to have more benefits for the upper back muscles such as the middle trapezius during lifting tasks (Goršič et al., 2022).

Muscle activity reductions were condition-specific during the *Dynamic* tasks (Figure 6). The peak moments provided by the exos are compared with these reductions in Supplementary Appendix E. In most cases, the two rigid exos reduced muscle activity with respect to the no-exo condition. Laevo only showed significant differences with no-exo conditions for the *Squat* task, and for unilateral erector spinae muscles during *Asymmetric* lifting. Paexo was most effective for the *Stoop* task and for some conditions in the *Asymmetric* task. For the *Squat* task, Paexo was not suitable and therefore not tested. Muscle activity reductions were on average 19.0 and 17.7% for the *Asymmetric* and *Stoop* tasks, respectively, which were comparable to findings in literature (Schmalz et al., 2022). Paexo is effective for stooping, although this movement results in higher low-back shear loads (von Arx et al., 2021).

Interestingly, the two soft exos were only effective during the *Squat* tasks in this study. Studies have shown variable findings in the task-specific nature of soft exos. Soft exos such as HeroWear Apex and VT-Lowe have shown significant reductions in muscle activity during squatting tasks (Lamers et al., 2018; Alemi et al., 2019; Goršič et al., 2021). Other studies show the effectiveness of HeroWear Apex and Corfor-V2 during stooped tasks (Lamers et al., 2020; Schwartz et al., 2021). PLAD was shown to be effective for both stoop and squatting tasks (Abdoli-E et al., 2006). Furthermore, another study even found that Auxivo encouraged stooped lifting when no strategy was enforced (Goršič et al., 2022).

None of the four exos had a significant influence on the Abdominal muscle activity as well as either oblique (Supplementary Appendix D). This is in line with literature (Kang and Mirka, 2023b; Reimeir et al., 2023; Schnieders et al., 2023), further strengthening the argument that passive back-support exos do not influence Abdominal muscle activity (Kang and Mirka, 2023b; Reimeir et al., 2023; Schnieders et al., 2023) during lifting tasks.

The participants in this study did not experience much discomfort when using the exos (Baltrusch et al., 2018; Kermavnar et al., 2021; Table 3). It is interesting to note that earlier versions of the Laevo were reported to have a high discomfort at the chest due to the previous chest pad design (Bosch et al., 2016; Alemi et al., 2020; Madinei et al., 2020). However, the Laevo Flex used in this study was found to have the lowest chest discomfort owing to the new chest harness. Overall, Laevo had low discomfort scores and was the only exo to show a significant reduction in perceived exertion with respect to the no-exo condition for the *Asymmetric* task. However, this reduction of 12% was lower than the average of 36% across passive exos reported in literature (Kermavnar et al., 2021). Despite larger reductions in muscle activity, Paexo did not show the lowest discomfort (Table 3) or reduced overall exertion (Table 4). Although both rigid exos reduced muscle activity during *Static* holding of the gear, the participants did not perceive lesser exertion when compared to no-exo condition (Table 4).

Among the four exos, the Darwing was perceived as the most comfortable, whereas Auxivo was perceived as the most uncomfortable. The discomfort experienced when wearing the Paexo and Auxivo could be due to the fitting of these exos (Goršič et al., 2022). The manufacturer recommends the leg and shoulder straps of the Auxivo be tightly fastened when standing in a neutral position. However, a few participants were too thin for the leg straps to be secured tightly. This resulted in the leg straps sliding upwards when the shoulder straps had to be tightened, resulting in friction in the groin. This is similar to the findings of Goršič et al. (2022). In the case of the Paexo, participants experienced discomfort at the front of the legs due to the design of the leg pads. These pads are made of inflexible material and have a specific curvature. This was not a good fit for all thigh circumferences, resulting in the highest discomfort at the front of the legs. Discomfort and restriction of movement must be addressed to avoid lower acceptance of exos in the workplace (de Looze et al., 2016; De Bock et al., 2022).

### 4.1. Limitations and future research

The study assessed two rigid and two soft exos for a given industrial workplace. Although we found results comparable to the literature, given the task-specific nature of the exos, care should be taken before generalizing to other exos or tasks (Alemi et al., 2020; Thamsuwan et al., 2020; De Bock et al., 2021). Moreover, the study has a limited number of participants and is underpowered.

The medium stiffness gas spring was used for the Laevo to provide similar torque assistance across participants. However, the manufacturer suggests varying the gas spring stiffness according to their sizing chart. This would result in variable assistance across participants (Van Harmelen et al., 2022). The impact of stiffness on reductions in muscle activity or user discomfort must be studied separately.

In this study, different approaches were used to estimate the assistive moments around the sacrum by the soft and rigid exos (Ali et al., 2021; Pesenti et al., 2021). These moments were estimated using a moment arm from the sacrum to the shoulder. A fixed moment arm was used to estimate the moments for the soft exos (Lamers et al., 2018; Moya-Esteban et al., 2023; Rodzak et al., 2023). This approach assumes that only tangential forces are exerted by the soft exos. Furthermore, it ignores the bending of the back and the resulting force distribution. Moreover, estimating the lumbosacral torques could provide better insight into the influence of the exos on low-back muscles (Abdoli-Eramaki et al., 2007; Lamers et al., 2018; Madinei et al., 2022).

Design and fitting of exos are very important to ensure intended assistance and comfort (Bhardwaj et al., 2023). The fitting for each of the four exos was done as close as possible to the manufacturer’s recommendations. However, multiple fitting and acclimation sessions are recommended to establish a perfect fit (Toxiri et al., 2019). Thus, conclusions drawn about the (positive) effects can only be improved when more time is spent on the fitting. Nonetheless, the fitting of the Auxivo could not be improved for some participants. The leg straps could not be tightened enough to tense the elastics optimally. Besides, the fitting of the Auxivo got worse with prolonged use. The straps loosened, and consequently, the elastic was tensed less and delivered less force. This would result in less assistance from the exo, and eventually lesser influence on muscle activity. Similar issues were found with the Paexo. Thus, these exos might require a redesign to improve their comfort.

Changes in muscle activity, comfort, and perceived exertion were not assessed during free movement in the laboratory. Thus, the impact of the exos on users during their intervals or breaks is not known. This may result in differences between the soft and rigid exos assessed. All participants were young adults and first-time exo users and were not a representative population of the industrial workplace that was being benchmarked. A broader study with varying population anthropometrics must be considered for the generalizability of results. Furthermore, we expected the participants to use their abdominal muscles as they were familiarized with the exo support. However, this was not the case for any exo. In any case, increasing familiarization with the exo could improve the interactions and its benefits.

A comprehensive benchmarking approach was utilized to assess suitable exos for the tasks in question. However, this may not be easily replicable in practice. Measuring the muscle activity of the erector spinae using EMG sensors requires extensive setup time, which is further complicated with the exo. Cable management was necessary to avoid hindrance in movement by the participant. Wireless EMG sensors may be a favorable alternative for exo studies; however, they are usually bulkier and may also cause discomfort when wearing the exo. Nonetheless, advances in measuring muscle activity in a wearable and less constricting and time-consuming manner are needed for future studies on assessing exos. Although the Hankamp Gears B.V. workflow was replicated in the laboratory, it must be complemented with a few extended studies at the actual workplace (Graham et al., 2009; Amandels et al., 2019; Motmans et al., 2019; De Bock et al., 2021). This can be enabled by using wearable setups consisting of inertial measurement units, pressure insoles, and wearable EMG sensors.

## 5. Conclusions

This study set up a benchmark to assess the use of four rigid and soft exos in a factory-inspired task. The study offers a novel comparison between assistive moments exerted by the exos and corresponding influences on muscle activity, comfort, and perceived exertion. Both rigid exos, Laevo and Paexo, offered the largest assistance during the *Dynamic* tasks. Overall, Laevo was found to have the best trade-off for reduction in muscle activity and comfort across *Static* and *Dynamic* tasks. Hence, the Laevo may be suitable during work where many different movements must be made. The two soft exos were most effective during *Squat* tasks, but as Auxivo shows the most discomfort, the Darwing is preferable. Given the task specificity of the exos across tasks, companies and users must assess the exos by benchmarking them for their workplace. We hope similar techniques are explored to evaluate newer exo designs across tasks. This will hopefully result in a better adoption of the back-support exoskeletons in the workplace and help reduce low-back pain.

## Supporting information

Mohamed Refai et al. supplementary material Mohamed Refai et al. supplementary material

## Data Availability

Data can be made available to interested researchers upon request by email to the corresponding author.

## Supplementary material

The supplementary material for this article can be found at https://doi.org/10.1017/wtc.2024.2.
