# Supplementary material for: Benchmarking commercially available soft and rigid passive back exoskeletons for an industrial workplace
Source: Wearable Technol. 2024 Feb 15;5:e6. doi: 10.1017/wtc.2024.2 (PMC10952052; doi:10.1017/wtc.2024.2)
Supplement: Mohamed Refai et al. supplementary material [file S2631717624000021sup001.pdf]

Appendices

Appendix A: Estimating forces and moments exerted by soft and rigid exos

Here, we explain the approaches used to estimate the forces and sagittal moments provided by soft and rigid exos during the *Dynamic* tasks.

Soft Exosuits

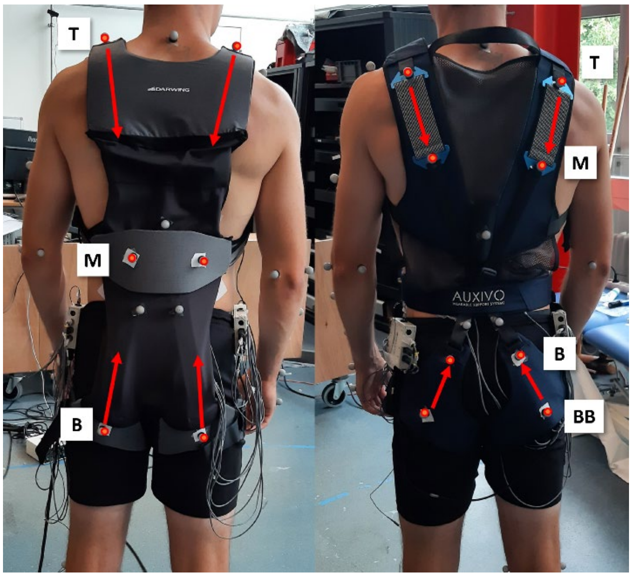

Fig. A.1. Soft exosuits apply forces parallel to the trunk. The direction and point of application of forces generated by the exosuit (red arrows). The stretch of the exos were measured using markers placed (red dots) for the Darwing (left) and Auxivo (right). In case of the Darwing, the elastic bands were 65% the distance between T and M markers.

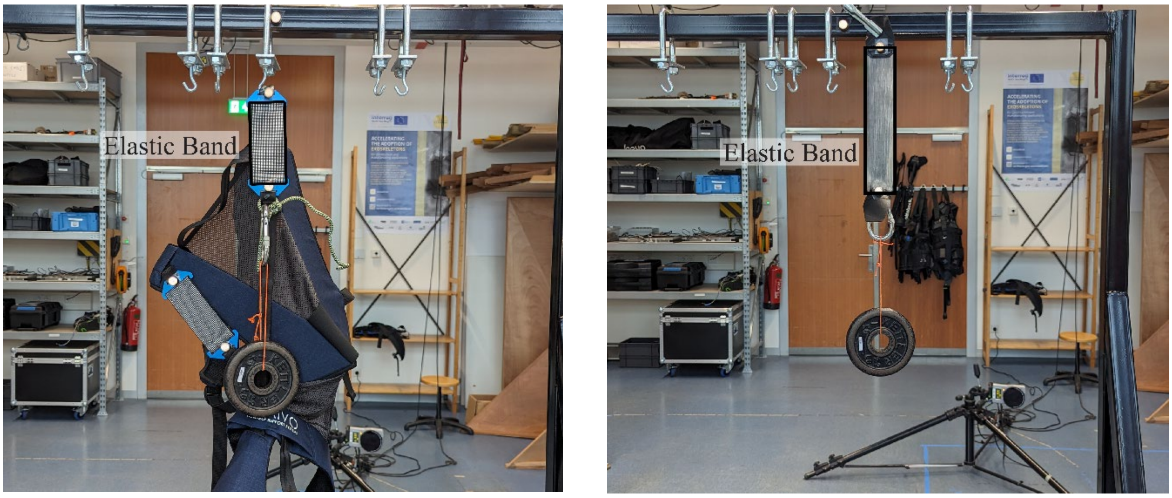

(a) (b)

Fig. A.2. The set-up used to determine the stiffness of the elastic bands of the exosuits. Elastic bands of (a) Auxivo and (b) Darwing were isolated. Loading and unloading curves were estimated for both bands with a set of weights. Markers were placed to find the elongation during the loading and unloading measurements.

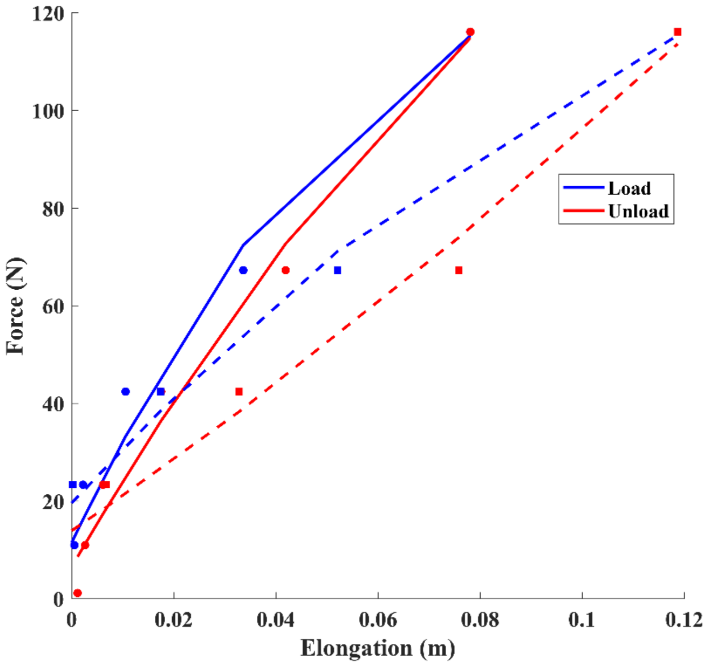

Fig. A.3. Force-elongation measurements for the elastic bands included in the Auxivo and Darwing. The circles and squares represent data points for the Auxivo and Darwing, respectively. A polynomial fit of order 2 was applied to these measurements resulting in the solid and dotted lines for the Auxivo and Darwing, respectively. Thus, each exosuit had two fits, one for loading and unloading of the elastic bands.

The soft exosuits used in this study (Fig. A.1) apply forces parallel to the human body due to the stiffness or damping offered by the exosuit. We estimated the force-elongation relation for each soft exosuit to estimate the forces during the dynamic task.

Figure A.2 shows the experiment setup to measure force-elongation relation. As we are interested in the forces acting on the upper body, we isolated the elastic bands that are present in the upper half of each exosuit. In case of the Auxivo, the elastic band is exposed, and can be loaded and unloaded directly. In case of the Darwing, the elastic band was isolated from the exosuit and tested. For both cases, loading and unloading of the bands was done with a set of weights including 0.12, 1.12, 2.38, 4.33, 6.86, and 11.83 kg. Markers were placed on the bands to measure the elongation. This resulted in respective force-elongation curves as seen in Fig. A.3. A polynomial fit of order 2 was applied to get the following equations for the Auxivo and Darwing respectively:

**Auxivo**	Loading	$F = 11.5 + 2178.2\,\Delta l - 10867\,(\Delta l)^2$
	Unloading	$F = 6.5 + 1801.7\,\Delta l - 5303.6(\Delta l)^2$
**Darwing**	Loading	$F = 19.6 + 1132.6\,\Delta l - 2743(\Delta l)^2$
	Unloading	$F = 13.9 + 706.7\,\Delta l - 1126.6(\Delta l)^2$

Here, $\Delta l_t = \left\|\mathbf{pos}_{\mathbf{T},t} - \mathbf{pos}_{\mathbf{M},t}\right\| - \left\|\mathbf{pos}_{\mathbf{T},1} - \mathbf{pos}_{\mathbf{M},1}\right\|$, where $\mathbf{pos}$ is the respective marker positions of $\mathbf{T}$ and $\mathbf{M}$ as seen in Fig A.1. The subscript (t and 1) denotes the time instance at which Δl is estimated. The force-elongation relations were used accordingly to estimate the forces acting on the user during the *Dynamic* tasks.

Rigid Exoskeletons

The rigid exoskeletons apply forces that are perpendicular to the body. The support moments provided by the exo at a given angle α was estimated from the measurement setup at Laevo B.V. (Rijswijk, The Netherlands) [1]. The setup measured torques during different α from the force exerted on a load sensor [1]. The torques due to gravity and the counter weight used were removed from the calculated torque to estimate the torque-angle relationship of the rigid exo. The torque-angle relationships were fit with polynomials of different orders to obtain the following relations:

**Paexo**	Loading	$M_\alpha = 5.7 + 1.8\,\alpha - 0.2\alpha^2 + 0.01\,\alpha^3 - (4.9 \cdot 10^{-4})\alpha^4 + (1.2 \cdot 10^{-5})\alpha^5 - (1.6 \cdot 10^{-7})\alpha^6 + (1.1 \cdot 10^{-9})\alpha^7 - (3.1 \cdot 10^{-12})\alpha^8$
	Unloading	$M_\alpha = 1.4 + 1.1\,\alpha - 0.1\alpha^2 + 0.01\,\alpha^3 - (4.3 \cdot 10^{-4})\alpha^4 + (1.1 \cdot 10^{-5})\alpha^5 - (1.4 \cdot 10^{-7})\alpha^6 + (8.9 \cdot 10^{-10})\alpha^7 - (2.3 \cdot 10^{-12})\alpha^8$
**Laevo**	Loading	$M_\alpha = -0.2 + 1.9\,\alpha - 0.002\alpha^2 - 0.002\,\alpha^3 + (3.2 \cdot 10^{-5})\alpha^4 - (2.8 \cdot 10^{-7})\alpha^5 + (8.4 \cdot 10^{-10})\alpha^6$
	Unloading	$M_\alpha = -1.1 + 0.9\,\alpha - 0.1\alpha^2 - 0.004\,\alpha^3 + (7.9 \cdot 10^{-5})\alpha^4 - (7.2 \cdot 10^{-7})\alpha^5 + (2.7 \cdot 10^{-9})\alpha^6 - (2.1 \cdot 10^{-12})\alpha^7$

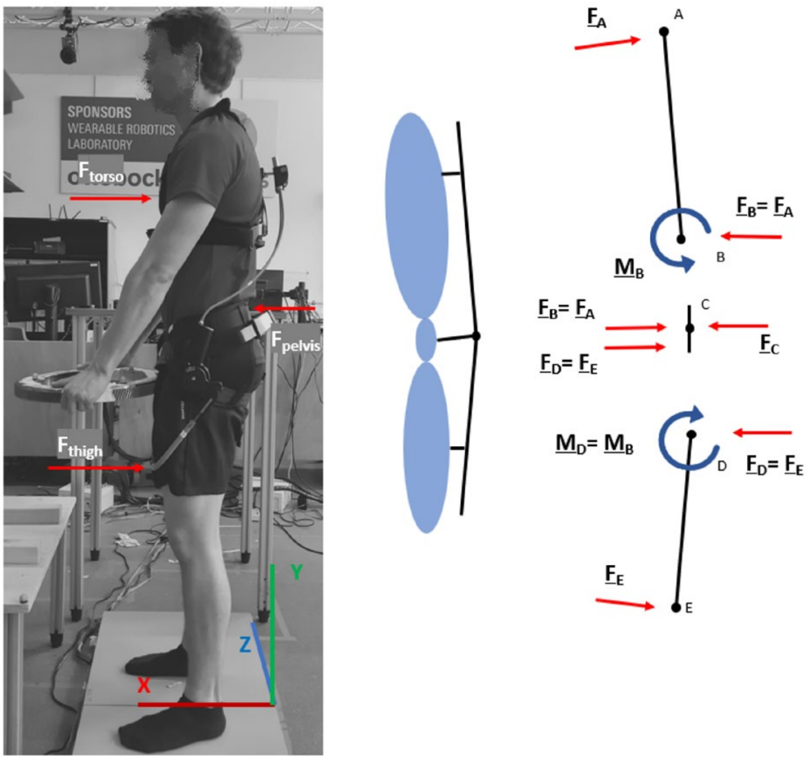

(a) (b) (c)

Fig. A.4. Forces of the rigid exoskeletons acting on the user. b) Schematic drawing of the human body (trunk, pelvis and thigh) and the exoskeleton. c) Free body diagram of the forces generated by the exoskeleton

During the *Dynamic* tasks, α was estimated as mentioned in Methods section 2.2.1. This was offset from the angle during static upright standing. The corresponding moment (M_α) was calculated from the torque-angle relationship using this angle. As a result of this support moment, the rigid exo applies forces at different points of contact on the user: the trunk, the pelvis and the thighs (Fig. A.4). A free-body diagram was drawn to account for the forces acting on the body due to the moment exerted by

the exoskeleton (Fig. A.4b and A.4b c). The moment can be resolved into three bilateral forces at the body: the trunk, the pelvis and the thighs. This results in moments ($\mathbf{M}$) at:

the trunk as $\mathbf{M}_{trunk} = (\mathbf{r}_{trunk}) \times \mathbf{F}_{trunk}$, and

the thigh as $\mathbf{M}_{thigh} = (\mathbf{r}_{thigh}) \times \mathbf{F}_{thigh}$.

Here, $\boldsymbol{r}$ is the distance between the hip joint (on the exoskeleton) and the point of application. The point of application on the trunk was defined between the C7 and T10 markers. For the thigh, the point of application was the marker on the leg pad of the exoskeleton. The trunk and thigh forces were assumed to be perpendicular to the trunk and thigh. Besides, it was assumed that the exoskeleton only transfers forces, not moments. Assuming that the moment M_α is planar, and using Varignon's theorem, the magnitude of forces at respective segments can be found as

$$F_{trunk} = \frac{M_\alpha}{\|\mathbf{r}_{trunk}\|},$$

$$F_{thigh} = \frac{M_\alpha}{\|\mathbf{r}_{thigh}\|}, \text{ and}$$

$$F_{pelvis} = -F_{torso} - F_{thigh}.$$

To split the trunk and thigh forces into the 3D components, two planes were defined to which the force was considered perpendicular. The plane for the trunk force was defined between the C7 and T10 and the left and right posterior superior iliac spine markers. The plane for the thigh force was defined between the marker on the exoskeleton joint, the medial femoral epicondyle and the lateral femoral epicondyle. Thus, 3D forces on the trunk were estimated as

$$\mathbf{F}_{trunk} = F_{trunk} \cdot \mathbf{u}_{trunk}$$

where $\mathbf{u}_{trunk}$ is the vector normal to the defined plane. Finally, $\mathbf{M}_{trunk}$ was estimated and the sagittal moments were identified.

Appendix B: Replicating the industrial workplace in the laboratory

The Computer Numeric Control (CNC) milling machine workplace at Hankamp Gears B.V. (Fig. B.1) was replicated in the laboratory with two tables (Fig. 3) of varying height. The working tasks of an employee was shadowed. This resulted in a list of *Static* and *Dynamic* tasks that resembled the daily tasks of the employee at the CNC machine. Snapshots of these are shown in Table B.1.

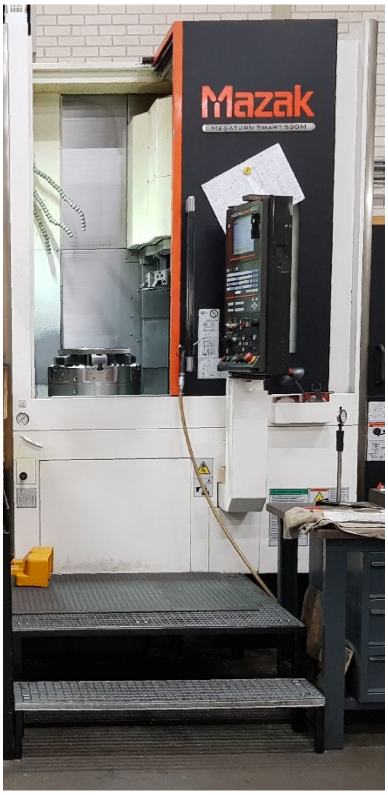

Fig. B.1. Photo of the CNC milling machine at Hankamp Gears B.V. The heights of the workplace was measured for replication of the setup in the lab.

TABLE B.1: SNAPSHOTS OF THE TASKS SIMULATING AN EMPLOYEE’S TASKS AT HANKAMP B.V.

Static tasks	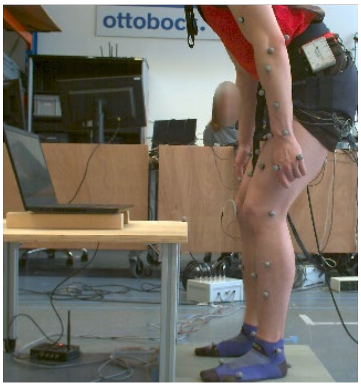 ***Static 40°***	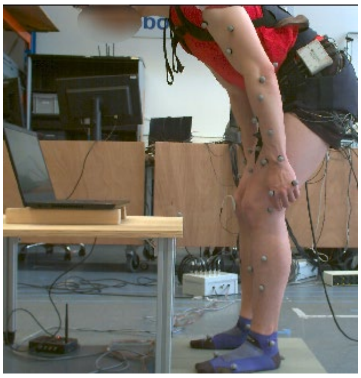 ***Static 60°***
Dynamic Tasks	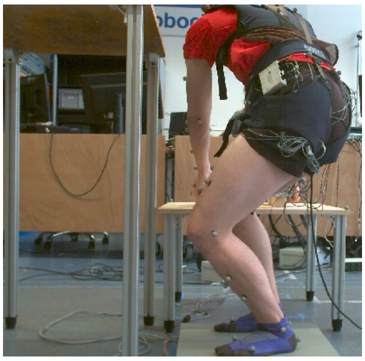 ***Asymmetric***	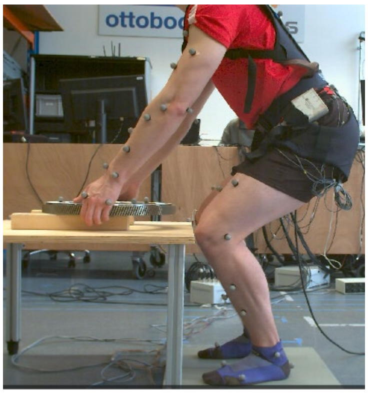 ***Squat***
	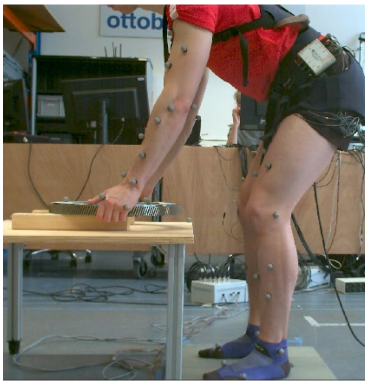 ***Stoop***	

Appendix C: Borg scale and VAS

The perceived rating of exertion (PRE) was based on the 15 points Borg scale (scale B) [2]. The lowest value was 6 corresponding to 'no sense of effort', and the highest possible value was 20 that represented 'maximum' effort by the participants. Table C.1 shows the English and Dutch versions of the scale.

The visual analogue discomfort scale was a range from 0 (no discomfort) to 10 (maximum discomfort) [3]. This was represented as a 10 cm along which the participants selected a point. The distance of this point from the start was measured and taken as the discomfort experienced. Figure C.1 shows the English and Dutch version of this scale.

The Dutch version of both scales were used during the experiments.

TABLE C.2: BORG SCALE B IN ENGLISH AND DUTCH.

Scale B	English	Dutch
6	No sense of effort	geen gevoel van inspanning
7	Extremely light	heel erg licht
8		
9	Very light	heel licht
10		
11	Light	licht
12		
13	Somewhat heavy	iets zwaar
14		
15	Heavy	zwaar
16		
17	Very heavy	erg zwaar
18		
19	Extremely heavy	extreem zwaar
20	Maximum effort	maximale inspanning

VAS Discomfort Scale

Global

	No discomfort	______________________________	maximum discomfort

Local

Chest	No discomfort	______________________________	maximum discomfort
Upper Back	No discomfort	______________________________	maximum discomfort
Lower Back	No discomfort	______________________________	maximum discomfort
Abdomen	No discomfort	______________________________	maximum discomfort
Front Legs	No discomfort	______________________________	maximum discomfort
Back Legs	No discomfort	______________________________	maximum discomfort

Globaal

	Geen ongemaak	______________________________	maximale ongemaak

Lokaal

Borst	Geen ongemaak	______________________________	maximale ongemaak
Bovenrug	Geen ongemaak	______________________________	maximale ongemaak
Onderrug	Geen ongemaak	______________________________	maximale ongemaak
Buik	Geen ongemaak	______________________________	maximale ongemaak
Voorkant bovenbenen	Geen ongemaak	______________________________	maximale ongemaak
Achterkant bovenbenen	Geen ongemaak	______________________________	maximale ongemaak

Fig. C.1. Visual analogue scale in English and Dutch. The participants received the Dutch version of the scale and made a cross along the line. The scale ranged from 0 (no discomfort) to 10 (maximum discomfort). The line was exactly 10 cm and the distance from the start was measured as the discomfort score. 10 cm line was offered to the participants.

Appendix D: Changes to muscle activity for the complete set of muscles

Here, we show the reductions in muscle activity across all muscle groups.

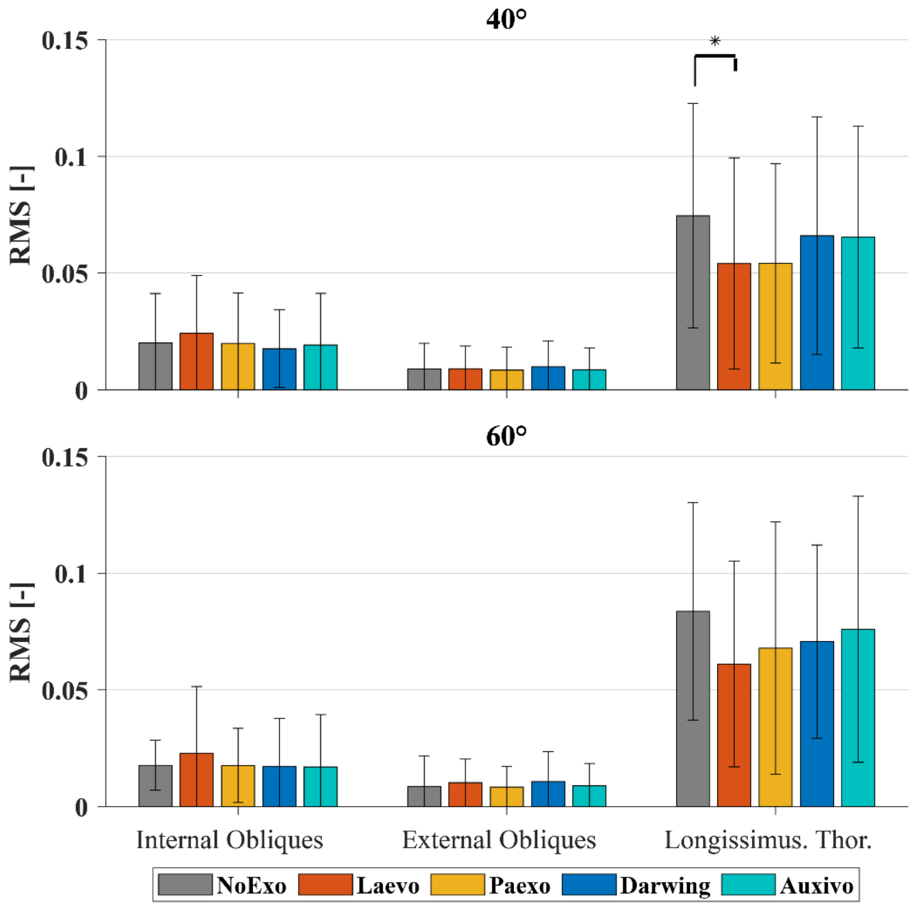

Fig. D.1. Median RMS of the normalised EMG during *Static* task across all participants. The Longissimus Thoracis muscle activity is shown here. Error bars represent the interquartile range. Significant differences are represented by the horizontal bars with *. The differences are read with respect to the left most condition (marked by the long thin line pointing down), and those that follow (shorter line pointing down).

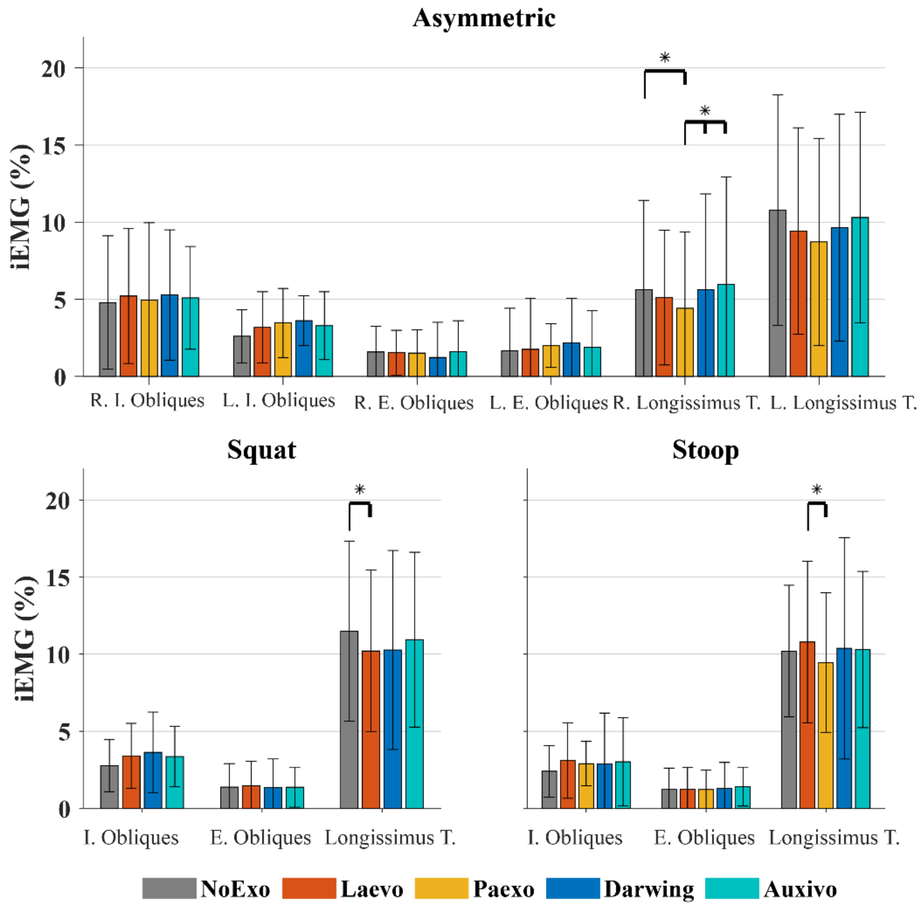

Fig. D.2. Median integral EMG of the *Dynamic* tasks across all participants. The Longissimus Thoracis muscle activity is shown here. Error bars represent the interquartile range. Significant differences are represented by the horizontal bars with *. The differences are read with respect to the left most condition (marked by the long thin line pointing down), and those that follow (shorter line pointing down).

Appendix E: Comparing exo assistive moments with reductions in muscle activity

TABLE E.1 EXO MOMENTS AND AVERAGE MUSCLE ACTIVITY REDUCTION WITH RESPECT TO NO EXO CONDITION.

Exo	Task	Peak Moments (Nm)	Average Moments (Nm)	Iliocostalis activity reduction (%)	Longissimus activity reduction (%)
Laevo	Asymmetric	33.8	15.8	29.9	18.1
	Squat	34.5	22.6	22.6	19.9
	Stoop	36.9	23.2	10.8	4.9
Paexo	Asymmetric	34.3	14.7	25.5	18
	Stoop	33.4	18.5	24.6	10.7
Darwing	Asymmetric	7.5	5.2	19.2	7.8
	Squat	7.2	5.4	16.6	10.8
	Stoop	7.8	5.6	-4.1	-1.7
Auxivo	Asymmetric	4.7	2.9	3.7	3.1
	Squat	3.8	2.7	14.2	13.7
	Stoop	4.2	2.9	9.1	-1.1

Table E.1 compares the assistance provided by each exo with the respective reduction in muscle activity compared to the no exo condition. In case of the *Asymmetric* task, the largest reduction across the left and right sides is shown. The muscle activity during the *Stoop* task for Darwing and Auxivo rather show an increase. The rigid exos are shown to have higher reductions in muscle activity during the *Asymmetric* task, whereas, the soft exos perform well for the *Squat* task.

REFERENCES

[1] V. Van Harmelen, J. Schnieders, and S. J. Wagemaker, “Measuring the amount of support of lower back exoskeletons,” no. October, pp. 1–5, 2022.

[2] G. A. Borg, “Nederlandse versie afkomstig uit KNGF-richtlijn Hartrevalidatie,” 1973.

[3] M. E. Wewers and N. K. Lowe, “A critical review of visual analogue scales in the measurement of clinical phenomena,” *Res. Nurs. Health*, vol. 13, no. 4, pp. 227–236, Aug. 1990, doi: 10.1002/nur.4770130405.
